# A systematic review of brain structural and functional alterations in pediatric mild traumatic brain injury

**DOI:** 10.3389/fnins.2026.1879109

**Published:** 2026-08-25

**Authors:** Hui Xu, Siqi Wu, Haocheng Zhan, Guanjinghui Xu, Junjie Zhao, Bo Yin

**Affiliations:** 1 Department of Neurosurgery, The Second Affiliated Hospital and Yuying Children's Hospital of Wenzhou Medical University, Wenzhou, Zhejiang, China; 2 School of Mental Health, Wenzhou Medical University, Wenzhou, China; 3 School of the 1st Clinical Medical Sciences (School of Information and Engineering), Wenzhou Medical University, Wenzhou, China

**Keywords:** functional connectivity, magnetic resonance imaging, neurodevelopmental trajectory, pediatric mild traumatic brain injury, white matter microstructure

## Abstract

**Introduction:**

Pediatric mild traumatic brain injury (pmTBI) constitutes 70–90% of childhood traumatic brain injury cases. While magnetic resonance imaging (MRI) reveals subtle brain alterations, fragmented evidence across modalities limits understanding of long-term neurobiological consequences.

**Methods:**

This systematic review synthesized 42 studies from MEDLINE/PubMed, Web of Science, Cochrane Library, and Scopus (inception to December 2025) employing structural MRI and functional MRI (fMRI). Methodological quality was assessed using a modified GRADE framework.

**Results:**

White matter investigations (*n* = 18) suggested widespread microstructural alterations in limbic and commissural pathways-alongside reduced global network efficiency and disrupted hub connectivity in executive and salience networks. Gray matter studies (*n* = 9) identified regional cortical thinning in prefrontal and parietal regions, hippocampal atrophy persisting up to 1 year. Resting-state fMRI studies (*n* = 5) revealed diminished default mode network (DMN) integrity and elevated striatal spontaneous activity. Task-based fMRI studies (*n* = 10) demonstrated compensatory hyperactivation during working memory and inhibitory tasks-manifesting as enhanced preparatory activation but resource depletion during execution-alongside lower DMN deactivation and persistent cerebrovascular reactivity abnormalities extending to chronic phases.

**Discussion:**

In sum, multimodal MRI provides evidence suggesting systematic structural and functional alterations in pmTBI encompassing white matter disruption, gray matter vulnerability, and network reorganization.

## Introduction

Mild traumatic brain injury (mTBI) represents an important and developing public health concern among children and adolescents worldwide. According to the global epidemiological statistics, the annual incidence rate of pediatric TBI ranges from 47 to 280 per 100,000 children, with mTBI constituting approximately 70–90% of these cases (Dewan et al., 2016; Schneier et al., 2006). In the United States, emergency departments record more than 640,000 annual visits for pediatric TBI (Bruns et al., 2021). The age distribution of pediatric mTBI (pmTBI) follows a bimodal pattern, with incidence peaks observed in children aged 0–4 years and adolescents aged 15–18 years, and a notable male predominance emerging after 3 years old (Dewan et al., 2016). Beyond the acute injury period, mTBI carries substantial implications for mental health outcomes. Depression represents a particularly concerning sequela, with reported rates ranging from 0 to 40% depending on assessment timing. Moreover, a scoping review highlighted that one longitudinal study reported 36% of children with mTBI screened positive for depressive symptoms on the CES-D within the first year following injury, compared to 12% of healthy controls (Sabir and Malhi, 2023).

In addition, cognitive function is affected with documented deficits in attention, executive function, working memory, and processing speed (Chadwick et al., 2021). Crucially, the developmental stage at injury significantly influences prognosis, as mTBI occurring during critical periods of brain maturation may disrupt neurodevelopmental processes and lead to cumulative functional deficits over time (Betz et al., 2024). This developmental vulnerability, coupled with substantial clinical heterogeneity, contributes to the troubling reality that approximately 25% of pediatric patients experience persistent post-concussive symptoms after the acute injury (Fried et al., 2022). Elucidating these complex interrelationships between injury characteristics, developmental context, and long-term outcomes is therefore essential for improving clinical care and reducing the substantial burden of pmTBI (Tuersunjiang et al., 2025).

To elucidate the neurobiological substrates of pmTBI, advanced multimodal magnetic resonance imaging (MRI) has become indispensable. Structural MRI (sMRI) encompasses assessments of gray matter morphological shifts, such as cortical thickness and volumetric alterations (Fried et al., 2022; Besteher et al., 2019), as well as white matter microstructural integrity quantified via diffusion-weighted imaging, including diffusion tensor imaging (DTI) metrics (Dolcos et al., 2020; Lindsey et al., 2019). Functional MRI (fMRI) includes both resting-state and task-based paradigms, which characterize alterations in functional connectivity within intrinsic networks such as the default mode network (DMN) and task-related activation patterns (Lindsey et al., 2019; Iraji et al., 2015; Xu et al., 2022). Collectively, these multimodal investigations suggest widespread structural and functional reorganization in pmTBI, which are linked to post-injury clinical symptoms and cognitive deficits (Lindsey et al., 2019; Rausa et al., 2020).

Despite the expanding body of neuroimaging literature in pmTBI, significant gaps remain that limit our understanding of its neurobiological impact. The evidence from a systematic review remains insufficient to draw definitive conclusions regarding the relationship between MRI findings and clinical outcomes (Rausa et al., 2020). This limitation stems from substantial methodological heterogeneity across studies, relatively small sample sizes, and inconsistent findings across imaging modalities-particularly evident in earlier systematic reviews that included only 22 studies with 448 participants and revealed inconsistent patterns of white matter alterations and functional connectivity changes (Schmidt et al., 2018). While a recent review specifically examined MRI modalities used in children and youth with persistent post-concussive symptoms and similarly highlighted substantial methodological heterogeneity and the absence of a clearly established MRI approach for detecting PPCS (Sheldrake et al., 2023), the present review synthesizes structural and functional MRI findings across the broader pmTBI population and across different post-injury phases, with PPCS considered as a clinical outcome rather than an independent eligibility criterion. Compounding these challenges, children represent a uniquely vulnerable population due to the dynamic brain development characterized by active synaptic pruning, myelination, and frontotemporal cortical maturation (Serpa et al., 2021; Babikian et al., 2015). Unlike the mature adult brain, the developing nervous system operates as a “moving target,” therefore mTBI may not only disrupt established functions but also derail ongoing developmental trajectories, potentially causing delayed emergence of deficits or arrested development of complex cognitive abilities that manifest only as children maturing and facing increasing academic and social demands (Serpa et al., 2021; Vuori et al., 2020).

Given these critical gaps, this present systematic review aimed to provide an integrated synthesis of multimodal neuroimaging findings in pmTBI. Importantly, PPCS was considered only as a relevant clinical outcome within the broader pmTBI literature and was not used as an independent target population or eligibility criterion in this review. Specifically, this review systematically: (1) characterized gray matter morphological alterations, including cortical thickness and volumetric changes; (2) delineated white matter microstructural abnormalities using diffusion-weighted imaging metrics; and (3) elucidated functional connectivity alterations and task-related activation patterns via resting-state and task-based fMRI, respectively. By consolidating evidence across these neuroimaging modalities, this study sought to advance understanding of the neural mechanisms underlying pmTBI during critical developmental windows, thereby informing clinical assessment and rehabilitation approaches tailored to this vulnerable population.

## Methods

### Search strategy

A systematic literature search was conducted in MEDLINE/PubMed, ISI Web of Science, Cochrane Library, and Scopus from database inception to December 2025, with no additional publication date restriction apart from the search cut-off date. The search strategy combined terms related to Magnetic Resonance Imaging (or MRI) and pediatric mild traumatic brain injury (or pmTBI) (see Table 1).

**Table 1:** Search strategy for identifying articles on MRI studies in pmTBI.

Search strategy
1. Pediatric
2. Children
3. Mild traumatic brain injury
4. mTBI
5. Brain structure
6. Brain function
7. Neuroimaging
8. Magnetic resonance imaging
9. MRI
10. Children search terms: 1 OR 2
11. mTBI search terms: 3 OR 4
12. Neuroimaging search terms: 5 OR 6 OR 7 OR 8 OR 9

mTBI, mild traumatic brain injury; pmTBI, pediatric mTBI; MRI, magnetic resonance imaging.

### Inclusion criteria

Eligible studies were limited to English-language, peer-reviewed original research studies that used MRI to assess brain structural and functional alterations in pmTBI. Dissertations, theses, conference abstracts, reviews, editorials, and other non-peer-reviewed materials were not included. Studies were required to involve human participants and to include either a direct comparison between a pmTBI group and a control group, or a continuous analysis of injury severity, symptom burden, or other clinical measures. To ensure a focused review of methodologically comparable studies, we restricted inclusion to MRI-based investigations, encompassing both structural and functional MRI modalities. No restrictions were imposed regarding the specific MRI techniques or analytical approaches (e.g., voxel-based morphometry, diffusion tensor imaging, resting-state functional connectivity, task-based activation). Consistent with the commonly used pediatric age definition in biomedical literature, the pediatric population was considered as individuals aged 0–18 years old during study screening (Strouse et al., 2022). In the final included studies, the participant age range was approximately 6–18 years.

### Definition of pmTBI

The operational definition of pmTBI varied across the studies included in this review. In the present review, the target population was pediatric mild traumatic brain injury, and PPCS was not considered as a separate clinical subgroup or independent eligibility criterion. Most commonly, included studies adopted established clinical criteria for mild TBI, including a Glasgow Coma Scale (GCS) score of 13–15, loss of consciousness (LOC) < 30 min, and post-traumatic amnesia (PTA) < 24 h. The eligibility was not restricted to the acute phase; studies conducted at acute, subacute, or chronic post-injury time points were included when the original injury was identified as pediatric mTBI. In some studies, pmTBI was operationalized through alternative approaches, including recruitment based on specific injury mechanisms or clinical labels commonly used in the mTBI literature, such as sports-related concussion, motor vehicle accident, self-reported or parent-reported medical diagnosis of concussion, or clinical presentation with post-concussive symptoms when formal GCS documentation was unavailable. These terms were considered operational approaches used by the original studies to identify pediatric mTBI cases, rather than separate eligibility categories in this review.

### MRI measures

MRI enables non-invasive assessment of brain structure and function by measuring signals from hydrogen protons in response to magnetic fields. Structural MRI provides high-resolution images for evaluating brain morphology, including gray matter volume, cortical thickness, and white matter integrity. Functional MRI (fMRI) infers neural activity through blood-oxygen-level-dependent (BOLD) signals, either during task performance (task-based fMRI) or at rest (resting-state fMRI). Resting-state fMRI data are amenable to multiple analytical approaches, including functional connectivity (both static and dynamic), amplitude of low-frequency fluctuations (ALFF/fALFF), regional homogeneity (ReHo), and fractal dimension analysis.

### Outcome measures

The primary outcomes of interest were MRI-derived measures of brain structural and functional alterations in pmTBI related with controls and its associations with clinical variables (e.g., injury severity, symptom burden, time since injury).

### Selection of studies

**Figure 1:**
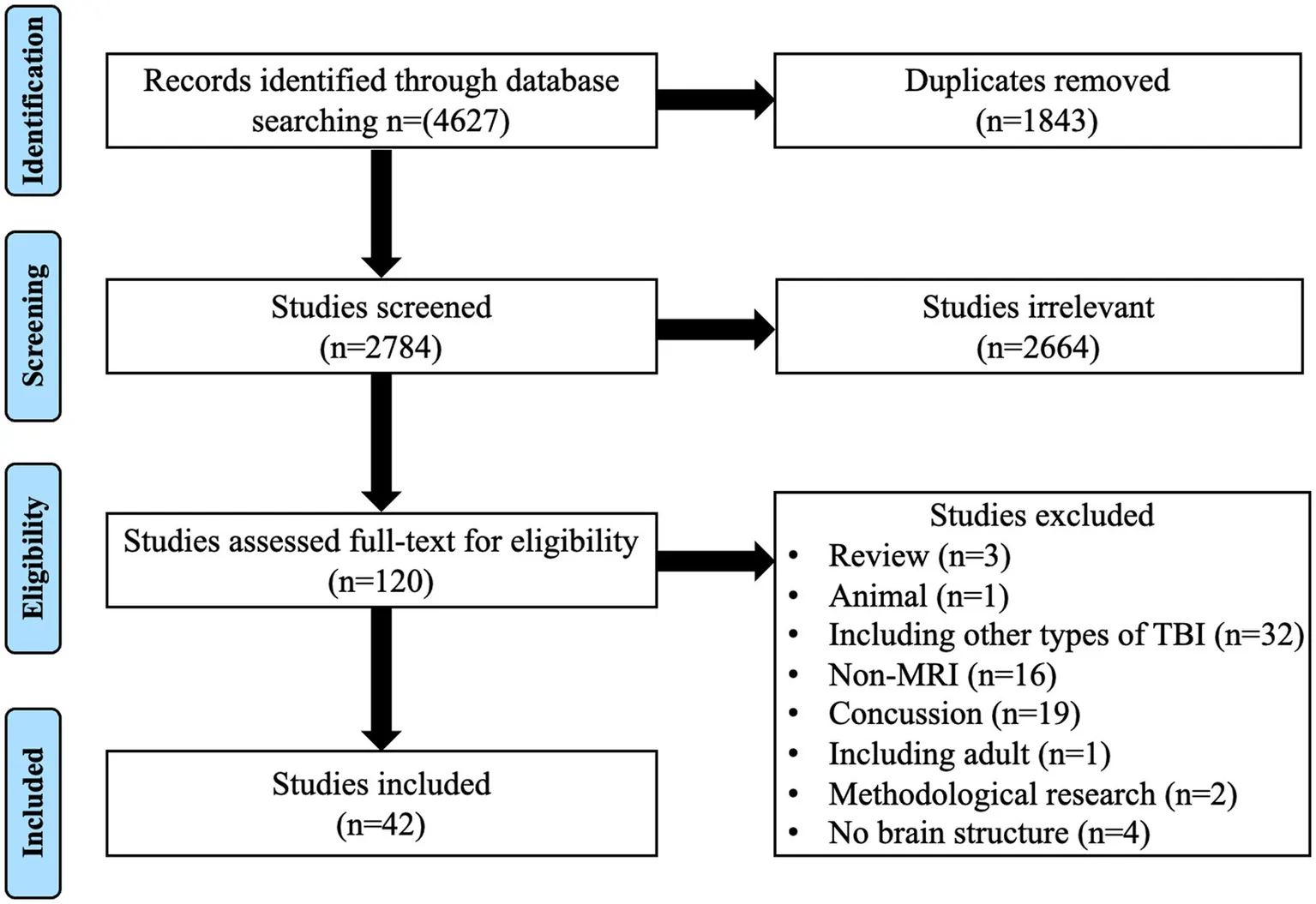
Preferred reporting items for systematic reviews and meta-analyses 2009 flow diagram of studies screened, included, and excluded in this review (Moher et al., 2009).

Prior to formal study screening, two independent reviewers completed a calibration exercise to ensure consistency in applying the inclusion criteria. Study selection followed a two-stage process. First, the same two reviewers independently screened titles and abstracts to identify potentially eligible articles based on predefined criteria. Any disagreements between the two reviewers were resolved through discussion or by consultation with a third reviewer. Second, full texts of potentially eligible articles were retrieved and independently assessed. Studies failing to meet the inclusion criteria at this stage were excluded. In total, 4,627 records were identified through database searching. After removing 1,843 duplicates, 2,784 records underwent title and abstract screening, of which 2,664 were excluded as irrelevant. The remaining 120 full-text articles were assessed for eligibility. Of these, 78 articles were excluded for the following reasons: review articles (*n* = 3), animal studies (*n* = 1), studies including other types of TBI (*n* = 32), non-MRI studies (*n* = 16), concussion-focused studies not meeting the eligibility criteria for pmTBI (*n* = 19), studies including adults (*n* = 1), methodological research (*n* = 2), and studies without relevant brain structure data (*n* = 4). Finally, 42 studies were included in this systematic review. The full screening process is presented in the PRISMA flow diagram (Figure 1) (Page et al., 2021).

### Data extraction and synthesis

After study selection, two independent reviewers extracted study-level information and reported findings from the included articles using a standardized electronic form. The extracted information included publication details (authors, year of publication, journal, and country), MRI modality (structural MRI, resting-state fMRI, task-based fMRI), verification of inclusion/exclusion criteria, sample size, participant demographic characteristics (age and sex), clinical characteristics of pmTBI (e.g., time since injury, injury mechanism, symptom severity, and Glasgow Coma Scale score when available), MRI data analysis approach (e.g., voxel-based morphometry, diffusion tensor imaging, functional connectivity, ALFF/fALFF, ReHo, and task activation contrasts), and the main brain structural or functional findings reported by each study. The extraction focused on study characteristics and reported neuroimaging results rather than raw participant-level data or quantitative effect-size data for meta-analysis.

Extracted findings were organized by MRI modality and outcome domain, including white matter microstructure, gray matter morphology, resting-state functional connectivity or spontaneous neural activity, and task-based functional activation. Within each domain, findings were summarized according to the imaging metrics used, brain regions or networks involved, direction of alterations, post-injury time frame, and reported associations with clinical symptoms or cognitive outcomes. Because the included studies showed substantial heterogeneity in MRI modalities, acquisition protocols, analytical methods, task paradigms, outcome measures, and post-injury assessment time points, a quantitative meta-analysis was not performed. Instead, we conducted a structured qualitative synthesis to identify recurring patterns, modality-specific findings, and inconsistencies across the included literature.

### Quality assessment

The methodological quality of included studies was appraised using a modified version of the Grading of Recommendations Assessment, Development and Evaluation (GRADE) framework (Guyatt et al., 2011). In this review, “quality” does not reflect a value judgment on the study itself, but rather refers to the pertinence of the study design and methods for addressing our specific review question regarding brain structural and functional alterations in pmTBI. Two independent reviewers evaluated each study across three domains (risk of bias, directness, and precision) using a standardized questionnaire developed for this review (Owens et al., 2019). For each domain, studies were rated as either “++” (exceeds criteria) if they met at least two domain-specific criteria, or “+” (adequately fulfills criteria) if they did not.

Risk of bias was defined as the presence of methodological factors that could lead to spurious or inflated findings, based on the following criteria: (a) Sample integrity: less than 20% of the total sample was excluded from analyses (e.g., due to poor image quality, excessive head motion). Exclusion of a high proportion of participants (>20%) may introduce selection bias and compromise generalizability. (b) Analytical approach: the study employed a voxel-wise, region of interest (ROI), or volume of interest (VOI) analysis. Voxel-wise or whole-brain voxel-based morphometry (VBM) approaches were considered most comprehensive as they allow unbiased, hypothesis-free comparison across the entire brain. ROI/VOI approaches, while valuable for testing specific hypotheses based on prior theory, were considered acceptable but required caution to avoid selective reporting (i.e., “cherry-picking” of significant regions). (c) Statistical rigor: the study implemented sufficient correction for Type I error (e.g., family-wise error correction, false discovery rate, Bonferroni correction). Given the large number of statistical comparisons inherent in neuroimaging data, studies without adequate correction for multiple comparisons were considered to have higher risk of bias.

Directness assessed the extent to which a study directly addressed our review question. Criteria for high directness included: (a) Population applicability: the sample comprised individuals with pure pmTBI. Studies that included mixed samples of mild and moderate/severe TBI were considered to have lower directness unless subgroup analyses for pmTBI were reported. (b) Confounder control: statistical control was implemented for potential confounding variables, including age, sex, intracranial volume, socioeconomic status, and comorbid psychiatric symptoms. (c) Appropriate control group: the study included a direct comparison between a pmTBI group and a control group, with groups matched on demographic factors (e.g., age, sex). (d) Group comparability: demographic variables were comparable between groups, or statistical adjustments were made for any baseline differences.

Precision referred to the accuracy and reliability of effect estimates. Given the heterogeneity of study designs in this literature, we developed study-specific criteria for assessing precision based on sample size, quality of exposure/outcome measurement, and imaging methodology. To ensure that the precision rating meaningfully discriminates between studies, we assigned greater weight to sample size, as the other two criteria are frequently met in neuroimaging studies and would otherwise result in most studies receiving the highest rating. Accordingly, a study was rated as “++” for precision only if it satisfied the sample size requirement (i.e., rated as high or moderate quality based on the criteria below) and met at least one of the two additional criteria (quality of exposure/outcome measurement or imaging methodology). Studies that met the sample size requirement but neither of the additional criteria received a “+” rating; studies that did not meet the sample size requirement received a “+” rating regardless of the additional criteria. Sample size requirements were defined separately for cross-sectional and longitudinal study designs, based on consensus among the review team regarding adequate statistical power for neuroimaging studies of pmTBI. Both “high quality” and “moderate quality” sample sizes were considered as meeting the sample size requirement for precision. For cross-sectional studies: High quality: ≥50 participants (single-center) or >100 participants across ≥2 centers with center effects accounted for; Moderate quality: 30–49 participants, or >100 participants across multiple centers without center effect correction; Low quality: <30 participants. For longitudinal studies: High quality: ≥35 participants enrolled at baseline (single-center) or >80 participants across ≥2 centers with center effects accounted for; Moderate quality: 20–34 participants enrolled at baseline, or >80 participants across multiple centers without center effect correction; Low quality: <20 participants enrolled at baseline. If the attrition rate exceeded 30% and no intention-to-treat analysis or missing data handling (e.g., multiple imputation) was performed, the rating was downgraded by one level. Priority rule for power analysis: If a study explicitly reported an *a priori* power analysis, the sample size rating was based solely on the percentage of the target sample achieved: ≥90% = high quality; 50–89% = moderate quality; <50% = low quality. Subgroup restriction: If the main conclusion of a study relied on a specific subgroup, the sample size of that subgroup was used for rating. Multi-site studies: multi-site studies that did not report or account for center effects were rated no higher than moderate quality, regardless of total sample size. In addition to sample size, precision was further informed by: (a) Quality of exposure/outcome measurement: Studies were considered to have higher precision if they used standardized, objective measures of mTBI severity (e.g., Glasgow Coma Scale score, loss of consciousness duration, post-traumatic amnesia duration). Moderate precision was assigned to studies using detailed symptom scales (e.g., Rivermead Post-Concussion Symptoms Questionnaire), while lower precision was assigned to studies relying solely on self-reported history of “mild concussion” without detailed verification. (b) Imaging methodology and quality control: Studies were considered to have higher precision if MRI acquisition parameters (sequence, field strength, resolution) were described in detail and remained consistent across all participants, and if image quality control procedures (e.g., motion correction, artifact removal) were reported.

Following quality appraisal, a narrative synthesis was conducted to summarize findings within each MRI modality (structural MRI, resting-state fMRI, task-based fMRI). The overall strength of evidence for brain structural or functional alterations in pmTBI was evaluated based on the aggregated ratings across the three GRADE domains.

## Results

Overall, 42 neuroimaging studies were included in this review. Of these studies, 18 studies utilized DTI and structural network analysis to investigate white matter microstructural alterations, revealing widespread changes in fractional anisotropy and diffusivity metrics across various tracts including the corpus callosum and cingulum bundle. Nine studies examined gray matter morphological characteristics through cortical thickness and volumetric analyses, identifying regional atrophy and altered developmental trajectories in frontal and temporal regions. Regarding fMRI, 10 task-based fMRI studies assessed working memory, inhibitory control, and reward processing, demonstrating both hyperactivation and hypoactivation patterns indicative of compensatory mechanisms or neuronal dysfunction. Additionally, 5 studies utilized resting-state functional connectivity and spontaneous activity metrics to explore DMN integrity, dynamic connectivity states, and cerebrovascular reactivity in pediatric mTBI populations.

### Investigations of white matter microstructure

In this domain, 18 studies investigating white matter microstructure in pmTBI, utilizing DTI, graph theoretical analysis, and neurite orientation dispersion and density imaging (NODDI) with cross-sectional and longitudinal designs. Among these, ten studies employing conventional DTI metrics reported significant alterations, with six studies demonstrating increased fractional anisotropy (FA) and/or decreased apparent diffusion coefficient (ADC), mean diffusivity (MD), and radial diffusivity (RD) in pmTBI compared to controls. Specifically, three studies identified elevated FA and reduced diffusivity within limbic network pathways including the fornix and cingulum bundle (Wu et al., 2010; Mayer et al., 2012; Yallampalli et al., 2010), while three additional studies observed similar alterations in commissural fibers (corpus callosum genu and splenium) and projection pathways (internal capsule, corona radiata, and superior longitudinal fasciculus) (Mayer et al., 2012; Van Beek et al., 2015a; Chung et al., 2019). Furthermore, four studies found significant associations between observed microstructural changes and clinical outcomes, including post-concussion symptom severity, memory dysfunction, and attentional deficits (Wu et al., 2010; Yallampalli et al., 2010; Chu et al., 2010; Madaan et al., 2021). Conversely, one large-scale investigation found no significant group differences in white matter metrics (Goodrich-Hunsaker et al., 2017).

In addition, 4 studies applied graph theoretical approaches, with 3 studies reporting altered network topology characterized by reduced global efficiency and disrupted hub connectivity in regions associated with executive control and salience networks, including the superior frontal gyrus, middle frontal gyrus, insula, and fusiform gyrus (Chung et al., 2019; Yuan et al., 2014; Ware et al., 2022; Ware et al., 2023a). One longitudinal study found that these topological abnormalities became more widespread at chronic timepoints (3–6 months post) compared to acute stages (Ware et al., 2023a). Additionally, one NODDI study revealed elevated orientation dispersion index (ODI) in the uncinate fasciculus and inferior fronto-occipital fasciculus among symptomatic children, which progressively normalized with clinical recovery and correlated with symptom severity (Stein et al., 2024). Longitudinal investigations also indicated persistent white matter abnormalities, with one study showing limited corpus callosum maturation over 6–8 months despite symptom resolution (Van Beek et al., 2015b), and another demonstrating time-dependent microstructural changes in anterior thalamic radiations among younger children with persistent symptoms (Ware et al., 2022) (Table 2).

**Table 2:** MRI studies investigating the white matter microstructure in pmTBI.

Study	Samples			Design	MRI	Main findings
	Subjects (case)	Definition of mTBI	Controls			
Wu et al. (2010)	Adolescents with mTBI, 6F/6M	A GCS score of 15 on arrival in the emergency department of Texas Children’s Hospital and had only brief loss of consciousness (<10 min).	5F/6M	Cross-sectional case control study	DWI/DTI	(1) Adolescents with acute mTBI exhibited significantly reduced ADC bilaterally in the cingulum bundles compared to controls.
						(2) FA of the left cingulum bundle was negatively correlated with 30-min delayed verbal recall in the mTBI group.
						(3) A trend was observed where reduced ADC in the left cingulum bundle was associated with poorer immediate verbal recall in the mTBI group.
Chu et al. (2010)	Adolescents with mTBI, 6F/4M	The initial GCS score was 15 for all mTBI	6F/4M	Cross-sectional case control study	DWI/DTI	(1) Adolescents with acute mTBI demonstrated significantly reduced ADC in multiple white matter regions and the left thalamus
						(2) The injury-affected regions exhibited decreased RD, unchanged AD, and increased FA.
						(3) Whole‑brain white matter DTI metrics detected abnormalities in acute mTBI that were associated with PCS.
						(4) Reduced ADC in white matter regions showed negative correlations with both PCS severity scores and emotional symptom scores (BSI).
						(5) The diffuse white matter alterations, particularly in frontotemporal regions and corpus callosum, suggest widespread axonal injury vulnerability in adolescent mTBI, while gray matter and CSF showed no significant group differences.
Yallampalli et al. (2010)	Adolescents with mTBI, 6F/5M	A brief loss of consciousness, an initial GCS score of 15 (as reported upon arrival at ED), and negative CT findings.	6F/5M	Cross-sectional case control study	DWI/DTI	(1) Adolescents with acute mTBI demonstrated significantly increased FA in the fornix.
						(2) Acute mTBI was associated with a trend of reduced ADC in the fornix, consistent with possible cytotoxic edema in the acute post-injury phase.
						(3) The mTBI group exhibited poorer processing speed performance on the ANAM, and higher fornix FA was significantly correlated with worse processing speed.
						(4) The negative correlation between fornix FA and processing speed was more pronounced in the mTBI group than in controls.
Mayer et al. (2012)	Pediatric mTBI, 2F/14 M	A closed head injury with GCS of 13–15, an alteration in mental status at the time of injury, a loss of consciousness 30 min, and posttraumatic amnesia limited to 24 h.	4F/12M	Prospective longitudinal case control study	T1/T2, DWI/DTI	(1) Semi-acute pmTBI exhibited widespread increases in fractional anisotropy (FA) and decreased radial diffusivity (RD) across multiple white matter tracts including the anterior corona radiata, cerebral peduncles, superior corona radiata, internal capsule, and corpus callosum, suggesting cytotoxic edema.
						(2) Lesion load metrics based on clusters of high anisotropy (number and volume) were significantly elevated in pmTBI and could objectively classify patients from healthy controls with 90% accuracy.
Yuan et al. (2014)	Pediatric mTBI, 2F/14 M	Sustained either a blow to the head or an injury with acceleration/deceleration movement to the head, a GCS score of 14–15 on presentation to the emergency department, and any one of the following: (i) loss of consciousness <30 min, (ii) amnesia, (iii) any alteration in mental state at the time of the injury.	4F/16M	Cross-sectional case control study	DWI/DTI	(1) Children with acute mTBI exhibited significantly higher small-worldness, normalized clustering coefficient, normalized characteristic path length, and modularity, along with lower global efficiency in global network topology compared to orthopedic injury controls.
						(2) Several hub regions, including the superior frontal gyrus, middle frontal gyrus, calcarine cortex, and precuneus, showed reduced nodal degree in the mTBI group.
						(3) The supplementary motor area and superior temporal gyrus temporal pole hubs demonstrated decreased nodal clustering coefficient in children with mTBI.
						(4) Nodal degree in the orbital superior frontal gyrus and middle frontal gyrus, nodal clustering coefficient in the lingual gyrus, and normalized betweenness centrality in the orbital superior frontal gyrus and calcarine cortex were correlated with post-concussion symptom scale scores.
Van Beek et al. (2015a)	Pediatric mTBI, 7F/13M	A pediatric GCS score of 13–15 and at least one of the following symptoms: (1) an alteration in mental status at the time of injury (e.g., confusion, disorientation, dizziness); (2) loss of consciousness <30 min; (3) post-traumatic amnesia <24 h; and/or (4) other neurological abnormalities that may or may not be transient (e.g., seizure or focal signs).	7F/13M	Cross-sectional case control study	DWI/DTI	(1) DTI analysis revealed subtle microstructural alterations in the corpus callosum (genu and splenium) of children with mTBI, characterized by higher FA and lower MD/RD, with no significant differences observed in the SLF or ILF.
						(2) The observed white matter abnormalities of the CC were not significantly associated with the observed mathematical difficulties in mTBI.
Van Beek et al. (2015b)	Pediatric mTBI, 6F/12M	A pediatric GCS of 13–15 and at least one of the following symptoms: (1) an alteration in mental status at the time of injury (e.g., confusion, disorientation, dizziness); (2) loss of consciousness<30 min; (3) post-traumatic amnesia <24 h; and/or (4) other neurological abnormalities that may or may not be transient	5F/13M	prospective longitudinal study followed the recovery from the sub-acute (1-month post-injury) to chronic stage (6–8 months post-injury) of recovery.	DWI/DTI	(1) The white matter abnormalities in the CC and mathematical difficulties observed in children with mTBI at the sub‑acute stage (1 month post‑injury) resolved after 6–8 months of recovery.
						(2) Longitudinal DTI data revealed impaired microstructural maturation of the damaged CC in mTBI children, evidenced by less pronounced increases in FA and decreases in RD compared to controls.
						(3) Children with mTBI showed higher FA values in the CC splenium compared to controls, indicating altered white matter integrity in this region.
Babcock et al. (2015)	Pediatric mTBI, 2F/21M	If they had either (i). a witnessed blow to the head, (ii). injury with acceleration/deceleration movement of the head, or (iii). self-reported injury with evidence of head trauma; a GCS score of 13 to 15 on presentation; and any one of the following: (i). loss of consciousness < 30 min, (ii). amnesia, or (iii). any alteration in mental state at the time of the injury.	5F/15M	prospective observational case–control study	T1/T2, DWI/DTI	(1) Youth with acute mTBI exhibited significantly higher FA and AD in multiple WM regions, including the middle temporal gyrus, superior temporal gyrus, anterior corona radiata, and superior longitudinal fasciculus, indicative of restricted water diffusion and potential cytotoxic edema.
						(2) The mTBI group demonstrated significantly lower MD and RD in several WM regions such as the middle frontal gyrus and anterior corona radiata, further supporting a pattern of restricted extracellular diffusion early post-injury.
						(3) Reduced RD in the right medial frontal gyrus WM and left corpus callosum was inversely correlated with acute symptom burden, suggesting a relationship between cytotoxic edema and symptom severity.
						(4) No microhemorrhages were detected on susceptibility-weighted imaging, indicating that axonal alterations can occur independent of microvascular damage in pediatric mTBI.
Goodrich-Hunsaker et al. (2017)	Pediatric mTBI, 31F/63M	Experienced head trauma resulting in hospitalization and who had a recorded day of injury post-resuscitation GCS score of 12 or less.	16F/43M	Cross-sectional case control study	DWI/DTI	(1) In pmTBI, increasing age is associated with elevated FA and reduced RD, MD, and AD across widespread white matter tracts, including the corpus callosum, cingulum cingulate, corticospinal tract, inferior and superior longitudinal fasciculus, and uncinate fasciculus.
						(2) Sex differences in white matter microstructure are observed in pediatric mTBI, with boys showing higher RD and MD in the superior parietal section of the corpus callosum and differential FA, RD, MD, and AD in specific tracts such as the cingulum cingulate, inferior fronto-occipital fasciculus, inferior longitudinal fasciculus, thalamic radiation, and uncinate fasciculus.
						(3) Tractography methods (AFQ and TRACULA) demonstrate superior sensitivity and tract-specific localization for detecting age- and sex-related white matter microstructural alterations compared to voxelwise TBSS in pediatric mTBI.
Stojanovski et al. (2019)	Pediatric mTBI, 29F/34M	A reported history of TBI with no skull fracture or neurosurgical intervention that was associated with one or more of the following: loss of consciousness for 30 min or less, amnesia for 24 h or less, or new-onset, post-TBI headaches.	PMC, 31F/32M; TD, 34F/29M	Cross-sectional case control study	DWI/DTI	(1) Youth with mTBI demonstrated increased low FA and decreased high FA in SWM relative to both typically developing and psychopathology-matched controls.
						(2) In DWM, mTBI youth exhibited decreased high FA compared to both control groups, while low FA was elevated only relative to typically developing controls.
						(3) Frontal lobe SWM displayed the most pronounced low FA alterations in mTBI youth.
						(4) Slower processing speed on an attention task was positively correlated with the extent of low FA in SWM, with the strongest association observed in the parietal lobe.
						(5) The proportion of high FA voxels was greater in SWM than in DWM among mTBI youth, suggesting a distinct susceptibility or compensatory response in superficial white matter.
Chung et al. (2019)	Acute mTBI:1F/7M; Subacute mTBI:1F/5M; Chronic mTBI:1F/6M	A blunt, sports-related injury to the head resulting in either (1) alteration in mental status (including loss of consciousness, disorientation, or amnesia) or (2) any of the following symptoms that started within four hours of injury and were not present before the injury: headache, nausea, vomiting, dizziness/balance problems, fatigue, drowsiness, blurred vision, memory difficulty or difficulty concentrating.	1F/7M	Longitudinal case‑control study	T1, DWI/DTI	(1) Longitudinal analysis demonstrated significantly increased FA and decreased MD in deep grey matter over time, with white matter volume significantly larger at the chronic stage compared to controls.
						(2) Global network topology exhibited significantly decreased transitivity and global efficiency alongside increased degree over time, indicating reduced network segregation and integration.
						(3) The local subnetwork showed significantly increased FA, network density (NoE), and decreased MD and RD longitudinally, suggesting enhanced white‑matter integrity in peripheral regions.
						(4) The rich‑club subnetwork had significantly lower MD and RD in acute mTBI compared to controls, with mean number of streamlines (NoS) altered over time.
Madaan et al. (2021)	Children with mTBI, 22F/52M	(1) at least 1 of the following: confusion or disorientation, loss of consciousness (<=30 min), posttraumatic amnesia (<=24 h), and/or other transient neurologic abnormalities not requiring surgery, occurring because of nonpenetrating craniocerebral trauma, and (2) a GCS score of 13–15 after 30 min postinjury or later on presentation to health care.	17F/34M	Prospective observational study with case‑control comparison.	T2, DWI/DTI	(1) MRI abnormalities were detected in 26.3% of children with mTBI in the acute phase.
						(2) Right arcuate fasciculus MD exhibited weak positive correlations with Verbal Comprehension Index and Processing Speed Index.
						(3) Right uncinate fasciculus FA positively correlated with Verbal Comprehension Index, and this correlation remained significant after controlling for age.
Ware et al. (2022)	Pediatric mTBI, 30F/53M	At least one of the following three criteria: an observed loss of consciousness, a GCS score of 13 or 14, or at least one acute sign or symptom of concussion as noted by ED medical personnel on a standard case report form, such as posttraumatic amnesia, focal neurological deficits, vomiting, headache, dizziness, or other mental status changes.	OI, 19F/18M; TD, 18F/21M	Cross-sectional case control study	DWI/DTI	(1) While mTBI and OI groups did not differ on any of the global graph theory metrics in final models, both the mTBI and OI groups demonstrated lower local efficiency (Elocal) and clustering coefficient (Cp) than the TD group.
						(2) As compared to the TD children, children with mTBI and OI had lower DC and NE and higher characteristic path length (NLp) in the fusiform gyrus, and lower clustering coefficient (NCp) in the superior and medial orbital frontal gyrus, paracentral lobule, insula, and thalamus.
						(3) Traumatic injury (regardless of site) alters whole-brain and regional networks in children. Children with OI may serve as a more conservative comparison group than TD children for pediatric mTBI neuroimaging research.
Ware et al. (2022)	Pediatric mTBI, 138F/224M	At least one of the following three criteria: an observed loss of consciousness, a GCS score of 13 or 14, or at least one acute sign or symptom of concussion as noted by ED medical personnel on a standard case report form, such as posttraumatic amnesia, focal neurological deficits, vomiting, headache, dizziness, or other mental status changes.	88F/110M	Prospective longitudinal cohort study	DWI/DTI	(1) Longitudinal white matter microstructural alterations were observed in pediatric mTBI, characterized by elevated MD in the anterior thalamic radiations at 3 months post-injury and reduced MD in the arcuate fasciculus at 6 months post-injury relative to orthopedic injury controls.
						(2) Symptom status modulated white matter microstructure, with mTBI children without persistent symptoms exhibiting higher FA in the superior longitudinal fasciculus and lower MD in the arcuate fasciculus and superior longitudinal fasciculus, particularly at 6 months post-injury.
						(3) Younger children with persistent symptoms demonstrated elevated MD in the anterior thalamic radiations across time compared to those without persistent symptoms and orthopedic injury controls.
						(4) Time post-injury moderated group differences in MD of the anterior thalamic radiations, arcuate fasciculus, and superior longitudinal fasciculus, indicating dynamic white matter microstructural evolution over months after injury.
Mayer et al. (2022)	Pediatric mTBI, 83F/121M	Inclusion criteria were based on both American Congress of Rehabilitation Medicine (upper threshold) and Zurich Concussion in Sport Group (lower threshold), and included GCS > 13, LOC (if present) limited to 30 min, PTA (limited to 24 h), alteration in mental status at the time of injury or at least two new PCS.	73F/100M	Prospective longitudinal cohort study	DWI/DTI/NODDI	(1) Standard DTI revealed increased FA and decreased MD in pmTBI patients from subacute to 4 months post-injury, predominantly in visual cortex, precuneus, and temporal regions, indicating persistent microstructural alterations.
						(2) Multicompartmental models (NODDI) demonstrated reduced free water fractions in grey matter and elevated intracellular volume fractions in widespread grey and white matter regions, suggesting cellular swelling and decreased edema.
Lapointe et al. (2023)	Pediatric mTBI, 4F/5M	At least one of the following criteria, consistent with the World Health Organization definition of mTBI: an observed loss of consciousness, a Glasgow Coma Scale score of 13 or 14, or at least one acute sign or symptom of concussion as noted by emergency department medical personnel on a standard case-report form.	4F/2M	Prospective concurrent cohort study	DWI/DTI	(1) Children with mTBI exhibited reduced global absolute phase during rest and lower DLPFC coherence during the 2‑back working memory task compared to orthopedic injury controls.
						(2) Global absolute phase was negatively associated with FA, indicating that white matter microstructural integrity relates to cerebral hemodynamic phase synchronization.
						(3) DLPFC coherence showed a negative correlation with FA and a positive correlation with MD, linking reduced functional connectivity to alterations in white matter microstructure.
Ware et al. (2023b)	Pediatric mTBI, 137F/223M	Sustained a blunt head trauma and met at least one of three criteria per the World Health Organization definition of mild TBI. Delayed neurological deterioration (e.g., Glasgow Coma Scale score <13), neurosurgical intervention, loss of consciousness >30 min or post-traumatic amnesia >24 h were exclusionary criteria for the TBI group. Children with OI sustained blunt force trauma to an upper or lower extremity that met criteria for mild OI based on the Abbreviated Injury Scale (i.e., score <=4).	87F/109M	Prospective longitudinal dual‑cohort study	DWI/DTI	(1) Regional structural connectome alterations in insula, cingulate, parietal, occipital, and subcortical regions, demonstrated by changes in clustering coefficient, betweenness centrality, and efficiency, were observed in pediatric mTBI, with these differences moderated by time post-injury, biological sex, and age at injury.
						(2) Regional network parameter alterations became more robust and widespread at chronic timepoints (3 and 6 months post-injury) compared to the post-acute phase, particularly in children with persistent symptoms.
						(3) Post-acute regional network metrics distinguished mTBI from mild orthopedic injury and predicted symptom recovery at 1-month post-injury.
						(4) Sex-specific effects were evident, with females showing reduced clustering coefficient in multiple cortical and subcortical grey matter structures at chronic periods, while males exhibited distinct patterns of network alterations.
						(5) Increased regional and local subnetwork segregation (modularity) and reduced efficiency emerged over time after mTBI, persisting up to 6 months post-injury, especially in females and adolescents with persistent symptoms.
Stein et al. (2024)	Symptomatic mTBI, 46F/34M; Asymptomatic mTBI, 12F/20M	Defined according to the American Academy of Neurology criteria.	12F/9M	Prospective cohort study	DWI/DTI/NODDI	(1) Symptomatic children with mTBI demonstrated significantly increased whole-brain ODI compared to HCs at both 1 and 2–3 months post-injury, reflecting ongoing microstructural reorganization in WM.
						(2) The elevated ODI was most pronounced in the UF and IFOF, particularly among those with poor recovery trajectories, indicating tract-specific microstructural alterations.
						(3) Higher ODI in WM regions showed a moderate correlation with greater post-concussion symptom severity (PCSI-Y scores), linking microstructural changes to clinical symptomatology.
						(4) ODI at 1-month post-injury exhibited superior predictive power for clinical recovery at 2–3 months compared to FA and clinical predictors alone, with WM ODI providing the highest predictive accuracy.

mTBI, mild traumatic brain injury; F, female; M, male; ADC, apparent diffusion coefficient; FA, fractional anisotropy; GCS, Glasgow Coma Scale; ANAM, Automated Neuropsychological Assessment Metrics; PCSS, post-concussion symptom scale score; MD, mean diffusivity; SLF/ILF, superior and inferior longitudinal fasciculi; CC, corpus callosum; TBSS, tract-based spatial statistics; AFQ, Automated Fiber Quantification; TRACULA, TRActs Constrained by UnderLying Anatomy; SWM, superficial white matter; DWM, deep white matter; PMC, psychopathology matched controls; TD, typically developing controls; ED, emergency department; OI, orthopedic injury; Dc, degree centrality; Ne, nodal efficiency; LOC, loss of consciousness; PTA, post-traumatic amnesia; PCS, post-concussive symptoms; DLPFC, dorsolateral pre-frontal cortex; ODI, orientation dispersion index; AD, axial diffusivity; RD, radial diffusivity; RPCSQ, Rivermead Post Concussion Symptoms Questionnaire; DTI, diffusion tensor imaging; WM, white matter; CP, cerebral peduncles; ACR/SCR, anterior/superior corona radiata; IC, internal capsule; SFGorb_L, left superior frontal gyrus orbital part; MFG_R, right middle frontal gyrus.

### Investigations of gray matter morphology

In this domain, 9 structural MRI studies examined gray matter alterations in pmTBI. Among these, six studies reported cortical thickness and volumetric alterations. Specifically, lower cortical thickness in the left dorsolateral prefrontal cortex (DLPFC) and right inferior parietal lobule, regions associated with executive control and attention networks, was observed (Urban et al., 2017). Another study reported reduced thickness in the right rostral middle frontal gyrus (Mayer et al., 2023), whereas another observed increased left parietal cortical thickness relative to orthopedic injury controls (Ware et al., 2020). Additionally, bilateral frontal atrophy accompanied by left temporal, parietal, and occipital cortical atrophy at 4 months post-injury was identified (Mayer et al., 2015). It has found that frontal cortical thickening was associated with loss of consciousness or post-traumatic amnesia (Nathaniel et al., 2025).

Regarding subcortical structures, three studies examined hippocampal morphology. Hippocampal atrophy persisting up to 1 year post-injury was identified (Nathaniel et al., 2025), while reduced hippocampal volumes with recovery mediated by injury severity markers were observed (Mayer et al., 2023). Additionally, dose-dependent interference with hippocampal neurodevelopment, specifically within the CA1 subfield, was further demonstrated (Nathaniel et al., 2025).

Clinically, 5 studies investigated associations between gray matter metrics and clinical outcomes. Right frontal cortical thickness was found to correlate with acute somatic PCS, whereas right temporal and inferior frontal thickness was associated with PCS (Ware et al., 2020). Increased parent-reported symptoms were reported to be associated with reduced cortical thickness in left inferior frontal and temporal polar regions (Bigler et al., 2018). Notably, structural alterations in prefrontal cortical regions and total cortical volume were associated with mental health outcomes, although formal mediation analyses have not consistently supported significant indirect effects (Lopez et al., 2022). However, two studies found no significant associations between structural abnormalities and PCS burden (Nathaniel et al., 2025; Dégeilh et al., 2023).

Longitudinal analyses revealed alterations in neurodevelopmental trajectories. It was demonstrated that mTBI alters typical cortical thinning courses, resulting in chronic cortical thinning up to 6 months post-injury, moderated by time, age, sex, and symptom status (Ware et al., 2023b). Similar developmental trajectories in social-brain regions between groups were found, except in the anterior temporal cortex where thickness remained stable (Dégeilh et al., 2023). Additionally, increased fractional anisotropy in the left temporal cortex (reflecting gray matter diffusion alterations rather than white matter fibers) and right thalamus during the subacute injury phase was observed (Mayer et al., 2015) (Table 3).

**Table 3:** MRI studies investigating gray matter morphology in pmTBI.

Study	Samples			Design	MRI	Main findings
	Subjects (case)	Definition of mTBI	Controls			
Mayer et al. (2015)	pmTBI, 2F/13M	A closed head injury with an alteration in mental status. GCS was 13–15 at the time of injury, loss of consciousness (if present) was limited to 30 min, and post-traumatic amnesia (if present) was limited to 24 h.	3F/12M	Prospective longitudinal case–control study	T1/T2, DWI/DTI	(1) Semi-acute pmTBI patients exhibited increased FA in the left superior temporal gyrus extending to the posterior insula and the right thalamus compared to controls, with decreased RD observed in the left temporal cluster.
						(2) The elevated FA in these gray matter regions persisted at approximately 4 months post-injury, particularly in the left temporal cortex.
						(3) Significant cortical atrophy emerged at 4 months post-injury in pmTBI, affecting bilateral frontal regions (superior and middle frontal gyri) and left-hemisphere areas including the middle temporal gyrus, postcentral gyrus, inferior parietal lobule, and cuneus.
						(4) FA abnormalities in both the left superior temporal gyrus and right thalamus accounted for variance in attentional functioning across cohorts.
						(5) No evidence of gray matter atrophy or subcortical volume reduction was present during the semi-acute injury phase.
Urban et al. (2017)	Children with mTBI, 13 M	Required a physician diagnosis in the past 3 to 8 months, and mTBI history was probed at study recruitment (using criteria by the 3rd International Consensus Conference on Concussion in Sport in Zurich (2008) and by the World Health Organization) and at the time of study participation (ThinkFirst Concussion Questionnaire created by Drs. J. Scott Delaney and Karen M. Johnston on behalf of the ThinkFirst-SportSmart Concussion Education and Awareness Committee).	14 M	Cross‑sectional case control study	T1	(1) Youth with mTBI exhibited significantly thinner cortices in the left DLPFC, right anterior IPL, and right posterior IPL compared to typically developing controls during the chronic phase (3–8 months post-injury).
						(2) The regions showing cortical thinning in mTBI youth overlapped with brain areas previously identified as having abnormal fMRI activation during dual-task performance.
						(3) The control group showed opposite associations where thicker left DLPFC and right IPL were associated with better working memory performance during single-task conditions, highlighting divergent brain-behavior relationships following mTBI.
Bigler et al. (2018)	Children with mTBI, 76F/143M	A GCS score> = 13 and met ACRM criteria for mTBI. Since this investigation focused on the mildest of injuries, the majority of the children with TBI had a GCS = 15.	32F/79M	Cross‑sectional case control study	T1	(1) In children whose injuries resulted from falls (combining both mTBI and OI groups), increased parental symptom reporting on the PCSI at 6 months post-injury was associated with reduced cortical thickness in the left inferior frontal, temporal pole, and lateral temporal lobe, as well as the right temporal pole.
						(2) The cortical regions showing thickness reductions in the fall group (temporal pole and inferior frontal areas) are implicated in emotional processing and pain perception.
Ware et al. (2020)	p mTBI, 45F/91M	In at least one of the following: (1) an observed loss of consciousness (LOC) for less than 30 min, (2) Glasgow Coma Scale (GCS) score of 13 or greater (GCS was 13 or 14 in cases with no LOC or other signs or symptoms), or (3) at least two acute signs or symptoms of concussion as noted on standard case report forms (e.g., post-traumatic amnesia, focal neurological deficits, skull fracture, vomiting, headache, dizziness, or other mental status changes).	24F/46M	Prospective concurrent cohort study with longitudinal follow‑up	T1	(1) mTBI group exhibited significantly greater cortical thickness in the left parietal region compared to the orthopedic injury group during the post-acute phase.
						(2) Age was negatively associated with cortical thickness in bilateral frontal, cingulate, parietal, and occipital cortices, indicating typical age-related thinning across both injury groups.
						(3) Greater right frontal cortical thickness was associated with higher parent-reported post-acute somatic post-concussive symptoms (PCS) across both groups.
						(4) Increased right cingulate cortical thickness was linked to higher child-reported post-acute somatic and 6-month cognitive PCS.
						(5) The association between right cingulate thickness and chronic cognitive symptoms was significant only in the orthopedic injury group, suggesting a differential relationship based on injury type.
Lopez et al. (2022)	possible mTBI, 200F/327M; mTBI, 80F/119M	Based on The Ohio State Traumatic Brain Injury Screen-Short Modified: Improbable TBI (no TBI or TBI without LOC or memory loss); Possible mild TBI (TBI without LOC but memory loss); Mild TBI (TBI with LOC < = 30 min)	5680F/6196M	Longitudinal cohort study	T1/T2	(1) mTBI is associated with reduced cerebrospinal fluid volume.
						(2) mTBI is linked to enlarged right inferior lateral ventricle volume in participants without incidental findings.
						(3) Whole brain volume is negatively associated with psychotic-like distress scores.
						(4) Total cortical volume is inversely correlated with both psychotic-like distress and total behavioral problems.
						(5) Bilateral hippocampal volumes are significantly smaller in children with higher psychotic-like distress and behavioral problems.
						(6) Reduced gray-white matter contrast in the dorsolateral prefrontal cortex is associated with greater psychotic-like distress.
Mayer et al. (2023)	pmTBI, 84F/124M	A closed head injury with GCS > =13, LOC (if present) limited to 30 min, PTA (if present) limited to 24 h, alteration in mental status at the time of injury, or at least 2 new PCSs.	74F/102M	Longitudinal cohort study	T1/T2	(1) Reduced cortical thickness in the right rostral middle frontal gyrus was observed in pmTBI patients compared to healthy controls.
						(2) Hippocampal volumes were significantly smaller in pmTBI patients relative to controls.
						(3) Hippocampal volume recovery was mediated by the presence of loss of consciousness (LOC) and/or posttraumatic amnesia (PTA), with patients reporting LOC/PTA exhibiting more persistent volume reduction at 4 months postinjury. I27
						(4) Structural abnormalities (cortical thickness and hippocampal volume) improved the specificity of classifying pmTBI patients versus healthy controls when combined with clinical measures.
						(5) Gray matter abnormalities, including cortical thinning and hippocampal volume reduction, may persist up to 4 months postinjury.
Dégeilh et al. (2023)	Children with mTBI, 145F/200M	Based on The Modified Ohio State University TBI Screen-Short Version: (1) Improbable TBI (no TBI or TBI without LOC or memory loss), (2) Possible mild TBI (TBI without LOC, but with memory loss), (3) Mild TBI (TBI with LOC 30 min)	3,627F/3,462 M	Longitudinal cohort study	T1/T2	(1) Children with mTBI displayed higher, albeit non‑clinical, levels of parent‑reported social problems at age 10, which decreased over the following 2 years but remained elevated compared to the stable trajectory observed in controls.
						(2) The variance of cortical‑thickness change in the left medial orbitofrontal cortex was significantly lower in the mTBI group than in controls, indicating reduced individual variability in the structural development of this social‑brain region.
						(3) Both mTBI and control children showed significant cortical thinning in multiple social‑brain regions (medial orbitofrontal cortex, banks of superior temporal sulcus, inferior parietal cortex) between ages 10 and 12 years, while anterior temporal cortical thickness remained stable in both groups.
Ware et al. (2023a)	pmTBI, 160F/247M	Sustained a blunt head trauma resulting in at least 1 criteria, consistent with the World Health Organization definition of mTBI: “(i) observed loss of consciousness, (ii) GCS score of 13–14, or (iii) at least 1 acute sign or symptom of concussion as noted by emergency department medical personnel on a standard case report form, including posttraumatic amnesia, focal neurologic deficits, vomiting, headache, dizziness, or other mental status changes.	94F/122M	Prospective longitudinal cohort study	T1	(1) pmTBI demonstrates chronic cortical thinning in region-specific patterns, with older children showing thinning in the angular gyrus and females exhibiting thinning in the basal forebrain, subcallosal area, and posterior orbital gyrus relative to mild orthopedic injury controls.
						(2) Symptom persistence after mTBI is associated with cortical thinning in frontal and occipital regions at 3 months postinjury in males and at 6 months postinjury in females and younger children compared to those without persistent symptoms and orthopedic injury controls.
						(3) Cortical thickness alterations after pediatric mTBI are moderated by time postinjury, age at injury, and biological sex across multiple brain regions, highlighting neurobiological heterogeneity linked to clinical symptom outcomes.
Nathaniel et al. (2025)	pmTBI, 112F/157M	With a Glasgow Coma Score > = 13, LOC (if present) limited to 30 min, PTA (if present) limited to 24 h, alteration in mental status at the time of injury, or the presence of at least 2 new post-concussive symptoms were enrolled. A semi-structured interview (the New Mexico Assessment of Pediatric Traumatic Brain Injury) was used to quantify LOC duration, PTA duration, alteration in mental status at the time of injury, or the presence of post-concussive symptoms.	104F/128M	Prospective longitudinal cohort study	T1/T2	(1) Presence of LOC/PTA is associated with increased brain age, frontal cortical thickening, and hippocampal atrophy up to 1 year after injury.
						(2) Mild head trauma interferes with hippocampal neurodevelopment in a dose‑dependent manner, including within the CA1 subfield.
						(3) Post‑concussive symptom burden is not associated with any structural abnormalities or alterations in neurodevelopment.

mTBI, mild traumatic brain injury; F, female; M, male; ADC, apparent diffusion coefficient; FA, fractional anisotropy; GCS, Glasgow Coma Scale; ANAM, Automated Neuropsychological Assessment Metrics; OI, orthopedic injury; PCS, post-concussive symptoms; LOC, loss of consciousness; PTA, posttraumatic amnesia; ED, emergency department; DLPFC, dorsolateral pre-frontal cortex; CA1, cornu ammonis subfield 1.

### Investigations of resting-state functional connectivity

A total of five resting-state fMRI studies were identified, investigating intrinsic brain function alterations in pediatric mTBI using various analytical approaches. Studies examining functional connectivity revealed that patients exhibited altered resting-state functional connectivity compared to controls, including both static and dynamic changes. Specifically, patients with persistent post-traumatic headache showed connectivity differences in affective pain processing regions such as the frontal lobe, temporal lobe, cerebellum, amygdala, and accumbens (Lemme et al., 2021). Dynamic functional connectivity analysis demonstrated that patients spent significantly less time in a frontotemporal default mode/limbic brain state at both subacute and early chronic phases (van der Horn et al., 2024). Static functional connectivity alterations included increased interhemispheric connectivity between bilateral precuneus and bilateral thalami, and decreased connectivity between the right supplementary motor area and left cerebellar area, with some changes persisting up to 1-year post-injury and correlating with executive function and memory deficits (van der Horn et al., 2024; van der Horn et al., 2025a). Using fractal dimension analysis, patients were found to have lower gray matter fractal dimension of resting-state blood oxygen level-dependent signals compared to controls, particularly in the right amygdala, cerebellar vermis, right caudate head, left hippocampus, and right hypothalamus (Dona et al., 2017). Furthermore, increased fractional amplitude of low-frequency fluctuations (fALFF) in the left striatum of patients at both subacute and early chronic phases was reported, indicating altered spontaneous neural activity, while regional homogeneity (ReHo) showed no group differences (Stephenson et al., 2020) (Table 4).

**Table 4:** PmTBI studies investigating resting-state functional connectivity.

Study	Samples			Design	MRI	Main findings
	Subjects (case)	Definition of mTBI	Controls			
Dona et al. (2017)	pmTBI, *N* = 15	Diagnosis of concussion based on Post-Concussion Symptom Scale (PCSS).	*N* = 56	Cross-sectional case–control study	T1, BOLD	(1) Overall grey matter fractal dimension (FD) of rs-BOLD signal was significantly lower in mTBI compared to controls. (2) Voxel-wise Z-score analysis identified specific brain regions with significantly reduced FD in individual mTBI patients, most frequently in the right amygdala, cerebellar vermis (culmen & uvula), right caudate head, left hippocampus, and right hypothalamus. (3) FD reduction in these regions aligns with previously reported dysfunction in mTBI and common neuropsychological symptoms. (4) Whole-brain grey matter mean Z-score showed a moderate positive correlation with total PCSS symptom score, though regional correlations were mostly low to moderate and non-significant. (5) Fractal analysis of rs-BOLD signal complexity shows potential as a patient-specific, non-invasive biomarker for detecting subtle functional disturbances in mTBI not visible on conventional anatomical scans.
Stephenson et al. (2020)	pmTBI, 58 M/47F	Based on American Congress of Rehabilitation Medicine and Zurich Concussion in Sport Group guidelines: closed head injury with Glasgow Coma Score ≥13, loss of consciousness ≤30 min (if present), posttraumatic amnesia ≤24 h, alteration in mental status at injury, or ≥2 new post-concussive symptoms.	62 M/51F	Prospective cohort study	T1/T2, BOLD	(1) pmTBI showed increased fALFF in the left striatum compared to HCs at both subacute (SA) and early chronic (EC) phases. (2) No significant group differences were observed for ReHo. (3) Contrary to hypothesis, no ReHo or fALFF abnormalities were specific to pmTBI with persistent post-concussive symptoms at 4 months postinjury. (4) Age-at-injury did not moderate ReHo or fALFF metrics.
Lemme et al. (2021)	pmTBI (PTH-P, 14F/7M; PTH-R, 11F/7M)	Youth meeting ICD-9 criteria for mTBI, diagnosed by a neurologist, who developed headache within 7 days of injury. Group assignment (PTH-P vs. PTH-R) was based on the persistence of headache symptoms beyond 1-month post-injury.	10F/10M	Cross-sectional investigation	T1	(1) Both PTH cohorts reported elevated fear of pain compared to healthy controls (HCs). (2) The PTH-P group showed altered resting-state functional connectivity (RS-Fc) relative to HCs in brain regions involved in affective pain processing (e.g., frontal, temporal, cerebellar, amygdala, accumbens). (3) The PTH-R group also showed altered RS-Fc (vs. HCs) between cerebellar and temporal lobe sub-regions, suggesting residual functional brain abnormalities even after symptom resolution. (4) Findings indicate symptom-level dependent patterns of brain network alterations in youth with PTH, implicating affective pain circuits in symptom chronicity.
van der Horn et al. (2024)	pmTBI, *N* = 200	Based on a combination of the American Congress of Rehabilitation Medicine for mTBI and the Zurich Concussion in Sport Group criteria: Glasgow Coma Scale 13–15, loss of consciousness ≤30 min, post-traumatic amnesia ≤24 h, alteration in mental status at injury, or at least two new symptoms immediately post-injury.	*N* = 179	Prospective cohort study	T1, BOLD	(1) pmTBI spent significantly less time in a frontotemporal default mode/limbic brain state (State 9) compared to controls at both subacute and early chronic visits. (2) pmTBI showed increased interhemispheric connectivity between left and right precuneus and between left and right thalamus, and decreased connectivity between the right supplementary motor area and left cerebellar area 10. (3) No significant associations were found between altered FC (dynamic or static) and post-concussive symptoms, injury severity measures (LOC, PTA, lesions), or history of previous mTBI.
van der Horn et al. (2025a)	pmTBI, *N* = 263 (1-week), 192 (4-months), 153 (1-year)	Pediatric mild TBI (pmTBI) diagnosed based on 1993 American Congress of Rehabilitation Medicine and Zurich Concussion in Sport Group criteria.	*N* = 228	Prospective longitudinal cohort study with three time points (sub-acute, early chronic, late chronic)	T1/T2, BOLD	(1) Persistent alterations in static functional connectivity (decreased SMA-cerebellar; increased thalamic) were observed in pmTBI vs. controls up to 1-year post-injury. (2) Precuneal static connectivity and fractional occupancy of a frontotemporal default mode/limbic dynamic brain state were altered at sub-acute and early chronic visits but normalized at 1-year, potentially due to pseudo-normalization from altered neurodevelopmental trajectories. (3) Altered precuneal connectivity was associated with lower executive function and long-term memory scores. (4) Findings suggest pmTBI results in chronic changes to intrinsic connectivity that interact with typical neurodevelopment.

EC, early chronic; fALFF, fractional amplitude of low-frequency fluctuations; F, female; FC, functional connectivity; FD, fractal dimension; HCs, healthy controls; M, male; mTBI, mild traumatic brain injury; PCSS, Post-Concussion Symptom Scale; pmTBI, pediatric mild traumatic brain injury; PTH, post-traumatic headache; PTH-P, persistent post-traumatic headache; PTH-R, resolved post-traumatic headache; PTA, post-traumatic amnesia; ReHo, regional homogeneity; RS-Fc, resting-state functional connectivity; rs-BOLD, resting-state blood oxygen level-dependent; SA, subacute; SMA, supplementary motor area.

### Investigations of task-based functional activation

Ten task-based fMRI studies were included, employing a range of paradigms to examine brain function in pmTBI during cognitive and physiological challenges. Specifically, greater bilateral posterior cerebellar activation during a combined working memory and inhibitory control task was observed in symptomatic children, with activation correlating with post-concussive symptoms (Krivitzky et al., 2011). Similarly, increased activation in left temporal, insular, and right frontal regions during high-load working memory in adolescents approximately 7.5 months post-injury was reported (Westfall et al., 2015). In contrast, it was found that symptomatic children at one-month post-injury showed decreased activation in precuneus, posterior cingulate, and left Broca’s area during a 1-back task compared to asymptomatic peers, with no differences from healthy controls, suggesting that activation patterns vary by symptom status and time since injury (Khetani et al., 2019).

Tasks probing cognitive control identified persistent alterations in response inhibition and proactive/reactive control. Hypoactivation in bilateral posterior cingulate, thalamus, basal ganglia, midbrain, and cerebellum during an Inhibition of Return task in subacute mTBI was found (Yang et al., 2012). Additionally, sustained altered brain activation in proactive response inhibition up to 4 months post-injury were demonstrated, characterized by hyperactivation during the cue phase and hypoactivation during the target phase in widespread brain regions including motor, sensory, and prefrontal cortex (Mayer et al., 2019). Moreover, lower DMN deactivation during both proactive and reactive cognitive control at the early chronic stage was observed, along with dynamic changes in cognitive control network activation (hypoactivation at subacute, hyperactivation at early chronic) (van der Horn et al., 2023).

Regarding spatial navigation, findings from a spatial navigation task showed symptom-level dependent activation patterns: low-symptom mTBI exhibited increased frontal and occipital activity, while the high-symptom group showed more widespread activation; both groups demonstrated reduced hippocampal and parahippocampal activity compared to controls (Holmes et al., 2018). Additionally, reward processing during a Monetary Incentive Delay task was examined in children with co-occurring conduct disorder and mTBI (Carr et al., 2025). The comorbid group displayed greater activation in the left amygdala and hippocampus during reward feedback compared to controls, conduct disorder-only, and mTBI-only groups. Studies using respiratory challenges further revealed persistent physiological alterations in cerebrovascular reactivity. Specifically, increased maximal fit and latency of end-tidal CO₂-regressed BOLD response in gray-white matter interface areas in patients at subacute and early chronic phases were found, with high classification accuracy (Dodd et al., 2020). Additionally, increased cerebrovascular reactivity amplitude in fronto-temporal and sensorimotor regions, increased latency in posterior midline regions, and reduced cerebral blood flow in bilateral cerebellar lobules VI/VIIa in patients up to 4 months post-injury were reported (van der Horn et al., 2025a) (Table 5).

**Table 5:** MRI studies investigating task-based functional activation in pmTBI.

Study	Samples			Design	MRI	Main findings
	Subjects (case)	Definition of mTBI	Controls			
Krivitzky et al. (2011)	pmTBI, 8 M/5F	Defined per CDC criteria: head impact resulting in brief mental status alteration, LOC < 30 min (or none), PTA < 24 h. All symptomatic at time of study (PCSI score >10). Excluded: prior concussion, significant medical/neurological history, IQ < 80.	7 M/6F	Cross-sectional case–control study	BOLD	(1) Both groups showed increased prefrontal activation with higher working memory load. (2) mTBI group showed greater bilateral posterior cerebellar activation during a task combining working memory with inhibitory control (1BI > 1B contrast) compared to controls. (3) No significant group differences on traditional paper-and-pencil neuropsychological tests. (4) mTBI group reported more post-concussive symptoms (PCSI) and real-world executive dysfunction (BRIEF). (5) In the mTBI group, greater post-concussive symptom report (PCSI) correlated with increased cerebellar and posterior cerebral activation during working memory tasks.
Yang et al. (2012)	pmTBI, 10 M/4F	Closed head injury, evaluated within 21 days (semi-acute phase). Inclusion per American Congress of Rehabilitation Medicine criteria: GCS 13–15 at initial assessment, alteration in mental status at injury, LOC < 30 min (if present), PTA < 24 h (if present). Excluded: history of neurological/psychiatric disorders, prior head injury with >5 min LOC, ADHD, learning disorder, substance abuse.	11 M/3F	Cross-sectional case–control event-related study	T1/T2, BOLD	(1) mTBI patients showed subtle, non-significant deficits on formal neuropsychological testing and during the Inhibition of Return (IOR) effect, with moderate effect sizes. (2) Functional imaging revealed mTBI patients had significantly decreased (hypo)activation in bilateral posterior cingulate gyrus, thalamus, basal ganglia, midbrain nuclei, and cerebellum compared to controls. (3) The pattern of hypoactivation was similar to that previously observed in adults with mTBI. (4) Activation levels in these regions correctly classified mTBI vs. controls with 85.7% accuracy. (5) fMRI was more sensitive to semi-acute mTBI effects than neuropsychological testing or structural imaging.
Westfall et al. (2015)	pmTBI, 13 M/6F	Children and adolescents 3–12 months post-concussion/mTBI, diagnosed by a sports medicine physician or qualified professional, confirmed using ACRM definition. Majority (17/19) had sports/recreational mechanism of injury.	10 M/9F	Cross-sectional case-control study	BOLD	(1) The mTBI group showed significantly greater brain activation than controls during the most difficult working memory load condition (2-back > 0-back), particularly in left temporal, insular, and right frontal regions. (2) There were no between-group differences in N-back task performance, neuropsychological test scores, or parent behavioral ratings (after correction for multiple comparisons). (3) Findings suggest compensatory brain activation to support normal task performance up to 1 year post-injury.
Holmes et al. (2018)	pmTBI (low/high symptom), 11F/16M	Diagnosed by a physician according to the World Health Organization (WHO) definition of mTBI. Recruited from the Concussion Clinic at The Montreal Children’s Hospital and the Montreal Neurological Institute.	9F/18M	Cross-sectional case-control study	T1, BOLD	(1) The low-symptom mTBI group (whose symptom levels did not differ from HCs) showed increased brain activity in frontal and occipital cortices during a spatial navigation task compared to HCs. (2) The high-symptom mTBI group reported significantly more symptoms than both HCs and the low-symptom group, and also showed an increased number of active brain regions. (3) Hippocampal and parahippocampal activity was significant in HCs but diminished in both mTBI groups, with further reduction in the high-symptom group. (4) Findings suggest mTBI patients exhibit unique, symptom-level dependent patterns of altered task-related brain activity, potentially indicating different compensatory mechanisms.
Khetani et al. (2019)	pmTBI (PPCS/recovered), 50F/40M	Children aged 8–18y presenting to ED within 14 days of mTBI, defined by AAN criteria (biomechanically induced brain function alteration with symptoms). PPCS defined as a 10-point increase in total Post-Concussion Symptom Inventory for Youth (PCSI) score and a 2-point increase in ≥2 symptom categories at 1-month vs. pre-injury.	10F/7M	Prospective controlled cohort study	T1/T2, BOLD	(1) At one-month post-injury, the symptomatic mTBI (PPCS) group showed decreased brain activation compared to the asymptomatic (recovered) mTBI group during the 1-back working memory condition, specifically in the precuneus, posterior cingulate, and left Broca’s area (DLPFC ROI). (2) No significant activation differences were found between either mTBI group and healthy controls. (3) Despite the brain activation differences, all three groups (symptomatic, asymptomatic, controls) performed similarly on the working memory task (accuracy and reaction time). (4) The findings suggest that altered cortical activation, particularly greater default mode network inhibition and reduced task-positive network activation, may underlie persistent symptoms even in the absence of performance deficits.
van der Horn et al. (2023)	pmTBI, *N* = 181	Based on American Congress of Rehabilitation Medicine and Zurich Concussion in Sport Group criteria: Glasgow Coma Scale 13–15, loss of consciousness ≤30 min, post-traumatic amnesia ≤24 h, alteration in mental status at injury, or ≥2 new symptoms immediately post-injury.	*N* = 162	Prospective cohort study	T1/T2, BOLD	(1) pmTBI showed reduced deactivation of the default mode network at the early chronic stage during both proactive and reactive cognitive control. (2) Hypoactivation in the cognitive control network (left pre-supplementary motor area) during proactive control at the subacute stage, with hyperactivation at the early chronic stage. (3) Alterations were more pronounced in the visual domain for proactive control and in the auditory domain for reactive control. (4) Task-related activations were not correlated with post-concussive symptoms.
Mayer et al. (2019)	pmTBI, 50 (59.5% M)	Pediatric mild traumatic brain injury (pmTBI) defined by closed head injury, GCS ≥ 13, alteration in mental status or ≥2 new symptoms, LOC < 30 min, PTA < 24 h, based on ACRM and Zurich guidelines.	*N* = 53	Prospective longitudinal cohort study with assessments at ~1 week and ~4 months post-injury.	T1/T1, pCASL, BOLD	(1) pmTBI showed persistent BOLD deficits in proactive response inhibition up to 4 months post-injury, with hyperactivation in motor and sensory cortices during cue phase (pmTBI>HC) and hypoactivation in motor, sensory, and prefrontal cortices during target phase (HC > pmTBI). (2) No abnormalities in resting-state functional connectivity or cerebral blood flow (CBF) were observed. (3) Clinical symptoms (PCS) improved but did not fully normalize; sleep disturbances and depression persisted. (4) BOLD abnormalities classified patients from controls with 75% accuracy, suggesting physiological recovery may exceed traditional recovery timelines.
Dodd et al. (2020)	pmTBI, 10F/20M	Pediatric mTBI (pmTBI) diagnosed based on a combination of the 1993 American Congress of Rehabilitation Medicine criteria and Zurich Concussion in Sport Group criteria.	14F/21M	Prospective longitudinal cohort study with assessments during sub-acute (∼1 week) and early chronic (∼4 months) phases of injury.	T1/T2, BOLD-CVR	(1) pmTBI displayed increased maximal fit/amplitude and increased latency to maximal fit of a time-shifted end-tidal CO2 regressor to BOLD response in grey-white matter interface areas throughout the brain, relative to HC. (2) These cerebrovascular reactivity (CVR) alterations persisted through the early chronic phase (∼4 months) of injury, outlasting the resolution of many clinical symptoms (e.g., post-concussion symptoms, headache, pain). (3) The maximal fit was associated with complaints of ongoing sleep disturbances at the early chronic phase but not with cognitive measures of processing speed or attention. (4) Increased maximal fit and latency to maximal fit together had high accuracy (90.8%) in classifying pmTBI from HC. (5) No group differences were found in resting cerebral blood flow (CBF) in regions with altered CVR.
van der Horn et al. (2025b)	pmTBI, 38F/69M	Pediatric mild traumatic brain injury (pmTBI) defined as closed head injury, GCS ≥ 13, LOC < 30 min, PTA < 24-h, alteration in mental status or ≥2 new symptoms, based on ACRM and Zurich guidelines, age range 8–18 years.	45F/65M	Prospective longitudinal cohort study with ~1-week and 4-month post-injury follow-up.	T1/T1, pCASL, BOLD	(1) pmTBI showed increased maximal fit of cerebrovascular reactivity (CVR) to end-tidal CO2 in fronto-temporal and sensorimotor regions (pmTBI>HC), and increased latency to maximal fit in posterior midline regions at both ~1 week and 4 months post-injury. (2) Reduced cerebral blood flow (CBF) was observed in bilateral cerebellar lobules VI/VIIa (HC > pmTBI). (3) A biphasic relationship existed between CVR amplitude and age (positive until ~14.5 years, negative thereafter) in both gray and white matter. (4) Neurodevelopmental changes did not moderate injury effects. (5) CVR and CBF abnormalities were not associated with post-concussive symptoms or cognitive deficits. Random forest analysis indicated cerebrovascular metrics classified pmTBI from HC with 67.1% balanced accuracy.
Carr et al. (2025)	pmTBI only, *N* = 1,216; CD only, *N* = 588; pmTBI+CD, *N* = 252	mTBI was defined based on parent-reported history of traumatic brain injury via the Modified Ohio State TBI Screen, categorized as improbable (no loss of consciousness or memory loss), possible mild (memory loss but no loss of consciousness), or mild (loss of consciousness <30 min). Cases with moderate or severe TBI were excluded.	*N* = 705	Cross-sectional study	BOLD	(1) During reward receipt (positive vs. negative feedback), mTBI and CD group displayed significantly greater activation in the left amygdala and left hippocampus compared to all other groups (TD, CD-only, mTBI-only). (2) The mTBI and CD group also showed increased activation in the right hippocampus compared to TD controls and the mTBI-only group, and in the right thalamus compared to TD controls. (3) No significant group differences were found in any of the eight regions of interest during reward anticipation. (4) The findings suggest that co-occurring CD and mTBI may be associated with unique hyperactivation in limbic regions during reward receipt, potentially reflecting enhanced emotional encoding of reward experiences, which may underlie increased reward-seeking behaviors and risk for delinquency.

AAN, American Academy of Neurology; ABCD, Adolescent Brain Cognitive Development; ACRM, American Congress of Rehabilitation Medicine; ADHD, attention deficit hyperactivity disorder; BOLD, blood-oxygen-level-dependent; BRIEF, Behavior Rating Inventory of Executive Function; CBF, cerebral blood flow; CD, conduct disorder; CDC, Centers for Disease Control and Prevention; CVR, cerebrovascular reactivity; DLPFC, dorsolateral pre-frontal cortex; ED, emergency department; F, female; fMRI, functional magnetic resonance imaging; GCS, Glasgow Coma Scale; HC, healthy control; HCs, healthy controls; IQ, intelligence quotient; IOR, Inhibition of Return; M, male; mTBI, mild traumatic brain injury; MID, Monetary Incentive Delay; PCS, post-concussive symptoms; PCSI, Post-Concussive Symptom Inventory; pmTBI, pediatric mild traumatic brain injury; PPCS, persistent post-concussive symptoms; PTA, posttraumatic amnesia; ROI, region of interest; TD, typically developing; WHO, World Health Organization.

### Quality assessment

The study quality of all included studies was evaluated using the GRADE. Regarding the Directness scale, all 42 studies were rated as good quality, meaning they directly tested whether pediatric mTBI patients showed distinct brain structural patterns compared to controls. Regarding the Precision scale, 31 studies were rated as good quality, meaning they used moderate or larger sample sizes, detailed clinical symptom scales or standardized objective assessments for mTBI severity, and had complete MRI scanning protocols with image quality control procedures; 11 studies were rated as fair quality due to insufficient sample sizes. Regarding the Absence of Bias scale, 41 studies were rated as good quality, meaning they carried out voxel-wise, ROI, or VOI analyses with proper adjustments for multiple comparisons; only one study was rated as fair quality because it excluded excessive samples in the analysis without sufficient Type I error correction, although it used ROI analysis (Table 6).

**Table 6:** Study details and quality assessment results.

Author(s), year	Reference	Absence of bias	Directness	Precision
Structural MRI-White Matter				
Wu et al. (2010)	*J Neurotrauma; 27: 303–307*	+ +	+ +	+
Chu et al. (2010)	*AJNR Am J Neuroradiol; 31: 340–346*	+ +	+ +	+
Yallampalli et al. (2010)	*J Neuroimaging; 23: 224–227*	+ +	+ +	+
Mayer et al. (2012)	*J Neurosci; 32: 17961–17,969*	+ +	+ +	+ +
Yuan et al. (2014)	*Hum Brain Mapp; 36: 779–792*	+ +	+ +	+ +
Van Beek et al. (2015a)	*J Neurotrauma; 32: 1567–1,578*	+ +	+ +	+ +
Van Beek et al. (2015b)	*Brain Inj; 29: 1701–1710*	+ +	+ +	+ +
Babcock et al. (2015)	*J Pediatr Rehabil Med; 8: 285–296*	+ +	+ +	+ +
Goodrich-Hunsaker et al. (2017)	*J Neurosci Res; 96: 626–641*	+ +	+ +	+ +
Stojanovski et al. (2019)	*Neuroimage Clin; 24: 102102*	+ +	+ +	+ +
Chung et al. (2019)	*Sci Rep; 9: 18833*	+ +	+ +	+
Madaan et al. (2021)	*J Child Neurol; 36: 664–672*	+	+ +	+ +
Ware et al. (2021)	*Hum Brain Mapp; 43: 1032–1,046*	+ +	+ +	+ +
Ware et al. (2022)	*Hum Brain Mapp; 43: 3809–3,823*	+ +	+ +	+ +
Mayer et al. (2022)	*Brain; 145: 4124–4,137*	+ +	+ +	+ +
Lapointe et al. (2023)	*Neurotrauma Rep; 4: 64–70*	+ +	+ +	+
Ware et al. (2023a)	*Brain Commun; 5: fcad173*	+ +	+ +	+ +
Stein et al. (2024)	*J Neurotrauma; 41: 41–58*	+ +	+ +	+ +
Structural MRI-Gray Matter				
Mayer et al. (2015)	*J Neurotrauma; 32: 723–730*	+ +	+ +	+
Urban et al. (2017)	*J Neurotrauma; 34: 816–823*	+ +	+ +	+
Bigler et al. (2018)	*Int J Psychophysiol; 132: 99–104*	+ +	+ +	+ +
Ware et al. (2020)	*J Neurotrauma; 37: 1892–1901*	+ +	+ +	+ +
Lopez et al. (2022)	*Neuroimage; 263: 119626*	+ +	+ +	+ +
Mayer et al. (2023)	*Neurology; 100: e516-e527*	+ +	+ +	+ +
Dégeilh et al. (2023)	*Cortex; 162: 26–37*	+ +	+ +	+ +
Ware et al. (2023b)	*Neurology; 101: e728-e739*	+ +	+ +	+ +
Nathaniel et al. (2025)	*Ann Neurol; 98: 341–353*	+ +	+ +	+ +
Resting-state fMRI				
Dona et al. (2017)	*PLoS One; 12: e0169647*	+ +	+ +	+ +
Stephenson et al. (2020)	*J Magn Reson Imaging; 52: 1701–1713*	+ +	+ +	+ +
Lemme et al. (2021)	*J Neurotrauma; 38: 1632–1,641*	+ +	+ +	+ +
van der Horn et al. (2024)	*Neuroimage; 285: 120470*	+ +	+ +	+ +
van der Horn et al. (2025a)	*Cortex; 184: 120–130*	+ +	+ +	+ +
Tasking-state fMRI				
Krivitzky et al. (2011)	*J Int Neuropsychol Soc; 17: 1143–1,152*	+ +	+ +	+
Yang et al. (2012)	*J Neurotrauma; 29: 2124–2,136*	+ +	+ +	+
Westfall et al. (2015)	*J Pediatr Rehabil Med; 8: 297–308*	+ +	+ +	+
Holmes et al. (2018)	*Brain Inj; 33: 383–393*	+ +	+ +	+
Khetani et al. (2019)	*J Neurotrauma; 36: 3274–3,283*	+ +	+ +	+ +
Mayer et al. (2019)	*Hum Brain Mapp; 40: 5370–5,381*	+ +	+ +	+ +
Dodd et al. (2020)	*J Cereb Blood Flow Metab; 40: 2491–2,504*	+ +	+ +	+ +
van der Horn et al. (2023)	*Hum Brain Mapp;44: 6173–6,184*	+ +	+ +	+ +
van der Horn et al. (2025b)	*J Cereb Blood Flow Metab; 45: 125–139*	+ +	+ +	+ +
Carr et al. (2025)	*Psychol Med; 55: e333*	+ +	+ +	+ +

+ + Denotes Exceeds Criteria. + Denotes Adequately Fulfills Criteria.

## Discussion

This systematic review of 42 multimodal MRI studies suggest that pmTBI may involved a complex interplay of structural damage, functional reorganization, and altered neurodevelopment. Structurally, pmTBI exhibited white matter (WM) microstructural alterations, disrupted network topology, and gray matter (GM) cortical thinning. Functionally, default mode network (DMN) integrity was compromised alongside compensatory hyperactivation during tasks. Crucially, the pediatric brain is not a scaled-down adult brain; it is dynamically remodeling. Consequently, regions undergoing peak development are highly vulnerable. This continuous maturation process leads to distinct pathophysiological mechanisms and imaging patterns in children, necessitating a developmental perspective when evaluating pmTBI.

Unlike adult with mTBI presenting decreased FA, pmTBI frequently showed acute FA increases. This likely reflects acute synaptic swelling, aberrant sprouting, or cytotoxic edema (Mayer et al., 2012). Over time, these metrics may decrease due to secondary degeneration or normalize via compensatory myelination. This temporal dynamism indicates that the pediatric brain reacts to injury by developing along a new, disrupted trajectory rather than settling into a static outcome. Moreover, graph theory revealed that pmTBI showed lower global network efficiency and disrupted hubs in the executive control and salience networks. These topological abnormalities often exhibited a temporal lag, becoming more widespread in the chronic phase and directly corresponding to clinical deficits in planning and attention.

In terms of GM, GM alterations are prominent in late-maturing regions (e.g., prefrontal cortex) and highly plastic areas (e.g., hippocampus) in pmTBI. Typical rate of cortical thinning was critically affected by pmTBI, leading to accelerated over-pruning or developmental arrest (Ware et al., 2023b). At the cellular level, this vulnerability is driven by post-injury excitotoxicity and an “energy crisis” that disproportionately impacts the high metabolic demands of the developing brain (Robertson et al., 2006). Notably, structural GM abnormalities do not always correlate with immediate symptom burden. This disconnect is likely due to the pediatric brain’s robust functional compensatory capacity, which can mask deficits until cognitive loads increase. Therefore, structural imaging must be paired with functional tasks to accurately capture brain-behavior relationships.

Integrating WM and GM evidence reveals that the essence of pmTBI is not static focal lesions, but a systemic interference with intrinsic developmental trajectories. Clinically, this means normal acute-phase imaging does not guarantee a favorable prognosis, as delayed developmental disruptions may emerge. Conversely, early structural abnormalities do not inevitably dictate poor outcomes, given the brain’s capacity for plastic compensation. Therefore, it’s critical to establish longitudinal monitoring. Future research must prioritize multi-center, longitudinal designs with extended follow-up windows, utilizing multimodal imaging to track multidimensional developmental trajectories. Investigating whether early interventions can guide the brain back to normative pathways will provide essential clinical targets. Ultimately, these structural trajectory deviations serve as the foundation for neural functional reorganization, which is essential for understanding altered information processing post-injury.

The sMRI reflects “hardware” damage, while the fMRI reveals the “software” reorganization of the brain. Post-injury functional alterations across core networks—including the default mode network (DMN), executive control network (ECN), salience network (SN) (van der Horn et al., 2023), limbic, and cerebellar systems—provide direct neural explanations for observed cognitive-behavioral deficits. The capacity of DMN to mediate transitions between internal and external attention is compromised. Patients exhibit reduced stability in resting-state networks and a critical failure to deactivate the DMN during cognitive control tasks. This inability to suppress mind-wandering systems clinically manifests as severe attentional lapses.

While pediatric patients often achieve normal behavioral scores, task-based fMRI reveals this comes at a high neural cost, due to the hyperactivation in ECN and SN to recruit additional resources (Westfall et al., 2015). Crucially, a dynamic coexistence of compensation and failure emerges: patients may hyperactivate during task preparation (cue phase) but deplete resources during execution (target phase) (Mayer et al., 2019). This “prepared but failing to execute” dynamic explains why children may perform adequately at times but suddenly experience cognitive fatigue or “go offline.” Reduced dynamic regulation in the limbic system (amygdala, hippocampus) strongly correlates with post-concussive symptom burden and emotional dysregulation. Furthermore, altered cerebrovascular reactivity extending to the cerebellum indicates impaired physiological regulation, potentially explaining the exacerbation of symptoms following physical exertion (van der Horn et al., 2025b).

Collectively, these alterations underscore three vital patterns: (1) a breakdown of dynamic equilibrium and synergy between networks [e.g., disrupted ECN-DMN antagonism (van der Horn et al., 2025a)]; (2) the high metabolic and neural cost of temporary compensation; and (3) symptom-network specificity, highlighting fMRI’s potential to provide objective neural markers for distinct clinical subtypes (e.g., altered limbic connectivity in persistent headache).

Though CT remained the primary acute imaging modality used for initial evaluation of mTBI in emergency settings, and even subtle CT findings such as pneumocephalus may carry prognostic significance (Hanalioglu et al., 2023), advanced MRI techniques can detect brain structural and functional alterations that exceed the resolution of conventional CT. Accordingly, MRI findings may complement, rather than replace, routine clinical assessment by providing additional insights into the neurobiological changes associated with post-injury symptoms and recovery trajectories among pmTBI.

From a practical perspective, these multimodal MRI findings in this review may provide additional neurobiological context for children with persistent cognitive, emotional, headache-related, or exertional symptoms, particularly when recovery does not follow the expected course. Practically, these findings may support closer monitoring, targeted neuropsychological evaluation, school-based accommodations, return-to-learn planning, and individualized rehabilitation. Prior studies have emphasized the importance of return-to-learn support after pediatric concussion (Master et al., 2012), and symptom-targeted rehabilitation, such as vestibular rehabilitation, may benefit selected adolescents after concussion or mTBI (Kontos et al., 2021). Future research should prioritize large, multi-center longitudinal studies with harmonized MRI protocols, standardized clinical and developmental outcome measures, and repeated assessments across acute, subacute, and chronic phases.

Several limitations should be acknowledged in this study. First, substantial clinical heterogeneity exists across studies including this review. Specifically, the wide age range across studies (6–18 years) entwines injury effects with normal developmental variance. Moreover, variations in mTBI diagnostic criteria and assessment timelines were observed in these studies. Second, substantial methodological heterogeneity exists across these studies including in this review, showing different structural analysis strategies, varied fMRI task paradigms, and heterogeneous brain functional metrics, and these heterogeneity prevents direct quantitative integration and meta-analysis of results in this review.

Despite the substantial clinical and methodological heterogeneity among the included studies, this review provides preliminary evidence for multi-level brain changes following pmTBI. Structural MRI indicates white matter microstructural perturbations, reduced network topology efficiency, regional gray matter vulnerability, and disruption of normal developmental trajectories. Functional MRI suggests deactivation deficits in the default mode network, dynamic coexistence of compensation and failure in executive control networks, and reduced regulation of the limbic system. These findings challenge the traditional notion of viewing children as “scaled-down adults,” emphasizing the need to establish longitudinal monitoring mechanisms in clinical assessment and to examine the potential impact of injury on long-term developmental trajectories. Future research should pursue multi-center, large-sample longitudinal studies to further evaluate the potential of imaging biomarkers into objective indicators for prognostic assessment.

## Data Availability

The original contributions presented in the study are included in the article/supplementary material, further inquiries can be directed to the corresponding author.
